# Effect of butorphanol nasal spray administration on patient cooperativity during labor epidural placement: a single-center randomized controlled trial

**DOI:** 10.1186/s13741-025-00535-7

**Published:** 2025-05-23

**Authors:** Jing Sun, Fan Wu, Mingguang Wu, Guanxiong Wu, Zhao Zheng, Gehui Li, Xiaoguang Wang, Xiaolei Huang, Yuantao Li

**Affiliations:** 1 Department of Anesthesiology, Shenzhen Futian District Maternal and Child Health Hospital, No. 2002 Jintian Road, Futian District, Shenzhen, 518000 China; 2 Department of Anesthesiology, Shenzhen Maternity & Child Healthcare Hospital, The First School of Clinical Medicine, Southern Medical University, Shenzhen, 518028 China; 3 Biomedical Research Institute, Hubei University of Medicine, No. 30 South Renmin Road, Shiyan, 442000 China

**Keywords:** Epidural block, Epidural puncture, Labor analgesia, Butorphanol nasal spray, Pain

## Abstract

**Background:**

Epidural block stands as the prevailing, secure, and efficient approach to labor analgesia. Inadequate maternal cooperation not only hampers anesthesia effectiveness but also may lead to severe consequences, including nerve damage due to positional changes.

**Methods:**

A randomized controlled clinical trial with 200 participants was conducted to compare painless delivery with epidural alone versus a combination of butorphanol nasal spray preceding epidural administration for painless delivery. The objective was to assess the combined approach’s efficacy in diminishing maternal pain and enhancing maternal compliance.

**Results:**

Within 8-min post-anesthesia, the combined analgesic group (EXP group) exhibited significantly lower maternal pain intensity scores, improved maternal cooperation, reduced visual analogue scale (VAS) pain, and McGill scores compared to the epidural alone group (CTRL group). No statistically significant differences emerged in 24-h postpartum blood loss, labor duration, or lactation period. Neonatal indicators, including umbilical artery blood PCO2, base excess of extracellular fluid (BE-ecf), weight, and Apgar score, showed no significant differences between the EXP and CTRL groups. However, the EXP group demonstrated a higher umbilical artery blood pH than the CTRL group. The EXP group exhibited significantly higher probabilities of pain intensity scores ≤ 6, maternal cooperation scores ≤ 3, VAS scores ≤ 3 at 6-, 8-, and 10-min post-anesthesia, and McGill scores of 0 compared to the CTRL group.

**Conclusion:**

Butorphanol nasal spray emerges as an effective means to alleviate pain during epidural puncture in labor analgesia, markedly improving maternal anesthesia adherence. This combined analgesic method proves to be a safe and efficacious approach for maternal pain relief during labor.

## Introduction

Epidural block stands as the foremost, secure, and highly efficient approach for providing analgesia during maternal labor (May 2003; Hawkins 2010; ACOG Practice Bulletin No 2019). Numerous women often find it necessary to seek analgesia when the intensity of labor pain is already considerable (Poma et al. 2019; Grant et al. 2015). Severe pain may lead many mothers to be uncooperative with the puncture position, facing risks such as spinal nerve injury and cerebrospinal fluid leakage, thereby limiting the application of epidural blocks. Simultaneously, achieving painless delivery through epidural blocks demands the continuous presence of an anesthesiologist in the delivery room around the clock. This not only requires their involvement in intense clinical operations (Bautista and George 2020) but also necessitates the provision of high-quality maternal labor analgesia. Consequently, the substantial workload on anesthesiologists further impedes the widespread adoption of epidural blocks, prompting concerns about maternal cooperation during spinal anesthesia and the burden on anesthesiologists.

Butorphanol, a mixed opioid receptor agonist primarily activating κ receptors with weak μ-receptor agonistic and antagonistic activity and minimal *δ* receptor activity, is notable for its role in modulating visceral pain, leading to effective relief (Craft and Mcniel 2003; Miller and Ernst 2004).

Butorphanol’s impact on the pain component of labor pain is significant, making it one of the rare drugs suitable for use during the maternal perioperative period, without increasing the incidence of opioid-induced adverse effects (Nelson and Eisenach 2005a). Despite its minimal passage through the placenta into the fetus, research has confirmed that butorphanol has minimal adverse effects on the fetus when administered to pregnant women, with rare instances of respiratory depression observed in newborn infants (Yadav et al. 2018). The noninvasive nature, ease of use, and high patient compliance of butorphanol nasal spray make it a favorable choice. Its rapid local absorption, circumventing first-pass metabolism in the liver, ensures high bioavailability (Wermeling et al. 2005). Several safety and efficacy of clinical trials have underscored the satisfactory analgesic effect of butorphanol nasal spray, with a swift onset of action post-nasal administration, a maintenance duration of 4–5 h, and a low incidence of adverse reactions (Desjardins et al. 2000; Abboud et al. 1991).

In light of the challenges related to maternal cooperation and the burden on anesthesiologists during spinal block anesthesia (Yadav et al. 2018; Nelson and Eisenach 2005b), this study explores the administration of butorphanol nasal spray in combination with spinal anesthesia for labor analgesia. The primary focus is to assess the safety of this combination and to ascertain its effectiveness in mitigating the issue of poor cooperation resulting from excessive pain in mothers undergoing spinal anesthesia. We predict that patients using butorphanol will significantly reduce labor pain during epidural puncture, thereby markedly improving maternal adherence to the epidural procedure.

## Methods

This prospective, double-blind, single-center randomized controlled trial enrolled 259 parturients undergoing labor between January 2020 and February 2021, exclusively involving tertiary hospitals affiliated with the Shenzhen Maternity & Child Healthcare Hospital. Ethical approval was obtained from the Ethics Committee of the Shenzhen Maternity & Child Healthcare Hospital, Southern Medical University, and the study was registered in the Chinese Clinical Trial Registry (ChiCTR2000032778). Informed consent was obtained from all participants.

Inclusion criteria are as follows:

1. Primiparas and multiparas classified as American Society of Anesthesiologists (ASA) II.
2. Single fetal head position >  = 37 weeks of gestation (including parturients and primiparas as well as parturients with spontaneous and oxytocin-induced labor). Maternal candidates with a need for labor analgesia and not currently using systemic analgesics.

Exclusion criteria are as follows:

1. Maternal allergy to butorphanol
2. Maternal receiving intravenous opioids during hospitalization or long-term oral opioids
3. Maternal diagnosed with fetal distress detected via heart rate monitoring
4. The presence of specific events after the study drug administration and before the seventh uterine contraction, including artificial rupture of the membrane, initiation of oxytocin infusion or a change in its rate, and full dilation of the maternal cervix within 2 h after administration. (This type of parturient progresses too rapidly for anesthesia to be administered in a timely manner).
5. Absolute and relative contraindications to neuraxial anesthesia and analgesia, such as patient refusal, puncture site infection, coagulopathy, uncorrected hypovolemia, increased intracranial pressure, severe cardiovascular stenosis, local anesthetic hypersensitivity, inadequate resuscitation medications, lack of first aid equipment, lack of patient cooperation, previous neurological deficits, spinal deformities or surgeries, and thrombocytopenia.

Throughout the trial, participants strictly adhered to obstetric anesthesia guidelines to minimize deviations, and subjects with puncture difficulty after three epidural attempts were excluded from the study.

### Method of analgesia

All parturient women undergoing observation in the delivery room underwent initial laboratory assessments, including blood type, hemoglobin levels, complete platelet count, and coagulation function evaluations. Continuous electronic fetal monitoring was maintained throughout the delivery process. Upon entry into the delivery room, maternal intake of solid food and high-energy, non-clear drinks were advised against. Obstetricians conducted evaluations to ensure maternal well-being and adherence to the study’s admission and exclusion criteria. Eligible participants, meeting these criteria, were included in the study after providing signed informed consent. The study employed a double-blind, randomized controlled trial (RCT) design. All parturients seeking labor analgesia were randomly assigned to two groups through computer-generated randomization. The experimental group (EXP) received inhaled butorphanol nasal spray combined with epidural labor analgesia, while the control group (CTRL) received epidural labor analgesia alone. Medications were prepared by anesthesiologists not involved in maternal management or evaluation procedures.

Parturient patients in the EXP group received one spray of inhaled butorphanol nasal spray, followed by another spray after 5 min. The single dose administered was 1.0 mg. Parturients in the CTRL group, with similar analgesic needs, received an inhaled saline nasal spray with the same appearance as the EXP group. Following administration, approximately 10–15 min later (after 6–7 contractions), the parturient was positioned left lateral, and epidural puncture was performed in the L2–3 gap following standard spinal canal puncture procedures. An epidural catheter was inserted, and lidocaine was administered followed by continuous monitoring. Additionally, a patient-controlled epidural analgesia (PCEA) pump was utilized, and vital signs were recorded 30 min after the first injection. Detailed recording of the extent of uterine orifice opening was performed, and all medications were ceased at the time of delivery. Additionally, during the labor analgesia process, if inadequate labor analgesia occurs, such as when the maternal pain score is greater than or equal to 5, a comprehensive assessment of the nature and location of the pain, as well as obstetric factors, should be conducted before implementing standardized management measures. These measures may include testing the level of epidural analgesia blockade, checking the position and depth of the epidural catheter, and ruling out any malfunctions in the drug infusion system (such as pump failure or catheter disconnection). Based on the manifestations of inadequate analgesia, potential causes should be considered, and appropriate interventions should be taken, such as administering 10 ml of 0.67% lidocaine combined with 0.3 µg/ml sufentanil. Detailed records of pain scores and vital signs should also be maintained.

### Degree of pain evaluation

Continuous monitoring of fetal heart rate and uterine contractions was implemented for all participants. Between the sixth and seventh contractions (occurring 10 to 15 min after inhaling the nasal spray), the mean pain intensity of the preceding two contractions was evaluated using a 0 to 10 visual analogue scale (VAS). Simultaneously, mothers were required to assess their pain levels and emotional status on the Short Form of the McGill Pain Questionnaire (SF-MPQ), and SF-MPQ consists of 15 pain description words, with 11 falling under the sensory category and 4 under the affective category. For items 1 to 11, related to physical pain, and items 12 to 15, pertaining to emotional status, each aspect was rated on a scale as follows: 0 = painless, 1 = mild, 2 = moderate, and 3 = severe. We have included all the data pertaining to the monitoring of nasal spray usage up to the completion of the epidural anesthesia, which lasted approximately 30 min.

### Assessment of the degree of cooperation of the maternal

Following the epidural procedure, anesthesiologists assessed maternal cooperation during the epidural puncture using a verbal score ranging from 0 to 10, where a lower score indicated a higher degree of maternal cooperation. Maternal analgesic levels were evaluated at the T10 level, and their VAS pain scores, along with modified Bromage motion scores, were recorded. Subsequently, adjustments to analgesic pump settings or drug concentrations were made based on each maternal’s pain level. Comprehensive records detailing maternal labor progress, fetal position, fetal conditions, and oxytocin use were meticulously documented.

### Ramsay sedation score

Sedation scores during the sixth and seventh contractions following the inhalation of butorphanol nasal spray were evaluated based on the following criteria. A score of 2–4 denoted good sedation, while a score of 5–6 indicated excessive sedation, providing a comprehensive assessment of sedation levels during labor.

1. Restless and irritable
2. Quiet cooperation
3. Lethargic, able to follow instructions
4. In a sleep state but can be awakened
5. In a sleep state unresponsive to strong stimuli
6. In a deep sleep and cannot be woken up

### Post-analgesia treatment

Following the aforementioned procedures, patients were transferred back to the delivery bed. Subsequently, midwives diligently monitored maternal contractions, fetal heart changes, and overall labor management. Timely responses to complications, including hypotension, slow heart rate, local anesthetic toxicity, and fetal heart rate deceleration, were administered. Interventions such as antihypertensive drugs in response to maternal heart rate, ephedrine for maternal hypotension and slow heart rate, and active management of uterine weakness by the obstetrician or midwife were implemented as required. The anesthesiologist adjusted the doses and concentrations of the local anesthetic in response to evolving conditions. In the event of fetal heart rate deceleration, immediate oxygen inhalation and meticulous adjustments to maternal position were carried out. The labor analgesia record sheet was duly completed. After delivery, the epidural catheter was removed, and mothers were transferred to the ward unless abnormal conditions were observed. Early follow-up and vigilant observation further complemented the post-analgesia care protocol.

### Primary outcomes

The primary outcome was maternal cooperation during epidural puncture, evaluated on a verbal score ranging from 0 to 10 by an anesthesiologist. Additionally, VAS pain intensity scores during epidural puncture (sixth and seventh contractions), and emotional scores assessed using McGill’s brief pain questionnaire, served as supplementary indices for evaluating maternal cooperation.

### Secondary outcome measures

Secondary measures included sedation scores, time intervals and contraction durations post-nasal spray inhalation, onset time of epidural labor analgesia, maternal satisfaction scores 24 h after delivery, delivery mode, delivery outcomes (on a scale of 0 to 10, representing “very dissatisfied” to “very satisfied”), Apgar scores at 1 and 5 min, and umbilical cord arterial blood pH values.

### Other observational index

General information, including age, gestational age, height, weight, BMI, parity, cervical dilatation (cm), complications, medication status, blood pressure, heart rate, and fetal heart rate (Philips Avalon FM20 dynamic monitoring, UK), was systematically recorded. Baseline pain intensity was assessed before nasal spray inhalation, and women rated the average pain of their last contractions on a 0–10 verbal scale. Emotional status related to pain was expressed by selecting a word from a provided list.

Contraction details during intrauterine pressure, time intervals, and duration post-nasal spray inhalation were monitored at various time points. Maternal uterine contractions, fetal intrauterine conditions, and the progress of labor stages were documented. The total dosage of epidural analgesic drugs, recovery drugs administered by the anesthesiologist, and the frequency of interventions were recorded. Other recorded information included maternal follow-up data and occurrences of adverse reactions within 24 h after delivery.

### Statistical analysis

Data analysis was conducted using R software (version 3.6.3). Continuous data were expressed as mean ± standard deviation (x̄ ± s). Measurement data conforming to normal distribution were compared using two-sided Student’s *T*-test, otherwise using Wilcoxon rank-sum test. Categorical data were presented as frequencies and percentages. Fisher’s exact test or Pearson’s chi-squared test was used to examine difference between the groups. The Shapiro–Wilk test was used to assess the normal distribution of the data. Univariate logistic regression identified potential risk factors. Multivariable logistic regression models were constructed based on significant factors, refined by Akaike information criterion (AIC)-based stepwise selection. The final model’s goodness of fit was assessed using the Hosmer–Lemeshow statistic. Pearson’s or Spearman’s rank correlation coefficient examined relationships between outcomes, with statistical significance set at *P* < 0.05.

## Results

### Participant and clinician flow

**Fig. 1:**
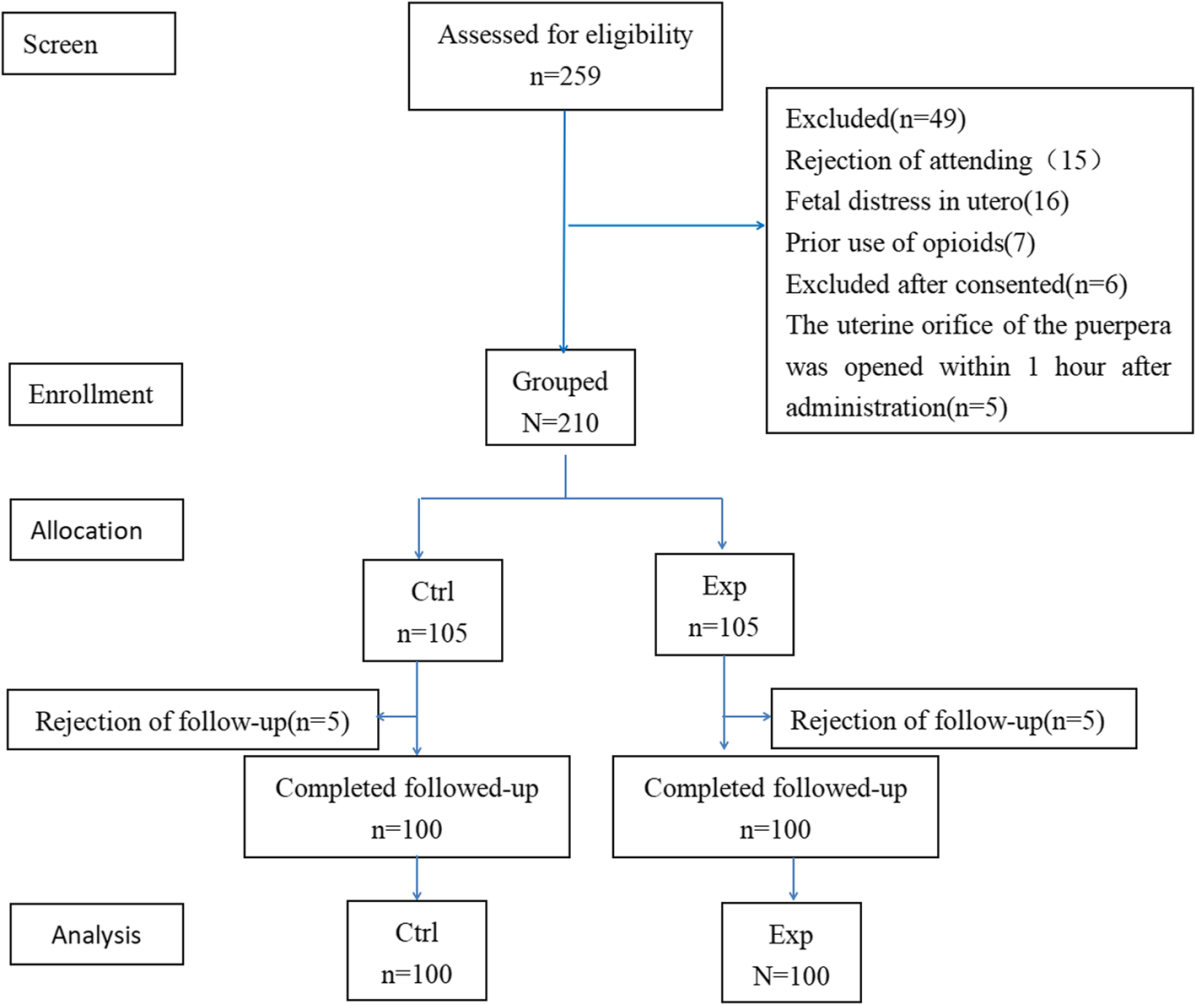
Flow of participants

The flow of participants throughout the trial is illustrated in Fig. 1. Of the 259 initially assessed patients, 15 cases declined participation, and 34 cases did not meet inclusion criteria (16 cases of fetal distress in utero, 7 cases with prior opioid use, 6 cases excluded after consenting, and 5 cases where the uterine orifice of the puerpera opened within 1 h after administration were excluded). A total of 210 participants were included initially, but 10 cases were lost to follow-up due to subsequent patient decline (5 in each group). Ultimately, 200 patients were included, evenly distributed between the experimental group (*n* = 100) and the CTRL group (*n* = 100) (Fig. 1). Based on relevant literature (Reference 20), a minimum of 66 cases is required when the standard deviation of the VAS score is 2, and a minimum of 146 cases is needed when the standard deviation is 3. Therefore, this study will encompass 200 patients, providing adequate power to discern the effective enhancement of maternal analgesia cooperation facilitated by butorphanol nasal spray. Baseline characteristics of participants in each group are detailed in Table 1. The groups demonstrated similarity across maternal age, gestational weeks, weight, height, BMI, cervical dilation, VAS score prenasal spray inhalation, and other parameters (Table 1).

**Table 1:** Baseline maternal demographic and obstetric characteristics

Characteristic	CTRL		EXP		Statistics	*p*-value
	***N***	***N* (%) or mean ± SD**	***N***	***N* (%) or mean ± SD**		
Mode of delivery						0.284^d^
Normal delivery	77	77 (77.00%)	84	84 (84.00%)		
Cesarean delivery	23	23 (23.00%)	16	16 (16.00%)		
Age (years)	100	31.63 ± 3.91	100	31.76 ± 3.64	4880	0.769^a^
Gestational weeks	99	39.12 ± 1.42	100	38.96 ± 1.41	5454.5	0.214^a^
Height (m)	100	1.61 ± 0.05	100	1.59 ± 0.05	5654	0.108^a^
Weight (kg)	100	68.03 ± 7.26	100	67.28 ± 6.80	0.750	0.454^b^
BMI (kg/m^2^)	100	26.41 ± 2.81	100	26.49 ± 2.78	4903	0.814^a^
Cervical dilation (cm)	100	2.36 ± 0.64	100	2.39 ± 0.69	4926	0.82^a^
VAS prior to inhalation of the nasal spray or saline	100	8.95 ± 0.38	100	8.91 ± 0.41	5236	0.445^a^
Parity					3.583	0.167^c^
1	13	13 (13.00%)	19	19 (19.00%)		
2	82	82 (82.00%)	71	71 (71.00%)		
3	5	5 (5.00%)	10	10 (10.00%)		
Hypertensive disorders of pregnancy	8	8 (8.00%)	4	4 (4.00%)		0.331^d^
Gestational diabetes mellitus	10	10 (10.00%)	9	9 (9.00%)		1^d^

^a^*P*-values were calculated by applying Wilcoxon rank-sum test

^b^*P*-values were calculated by applying two sample *t*-test

^c^*P*-values were calculated by applying Pearson’s chi-squared test

^d^*P*-values were calculated by applying Fisher’s exact test

### Primary outcome measures

**Fig. 2:**
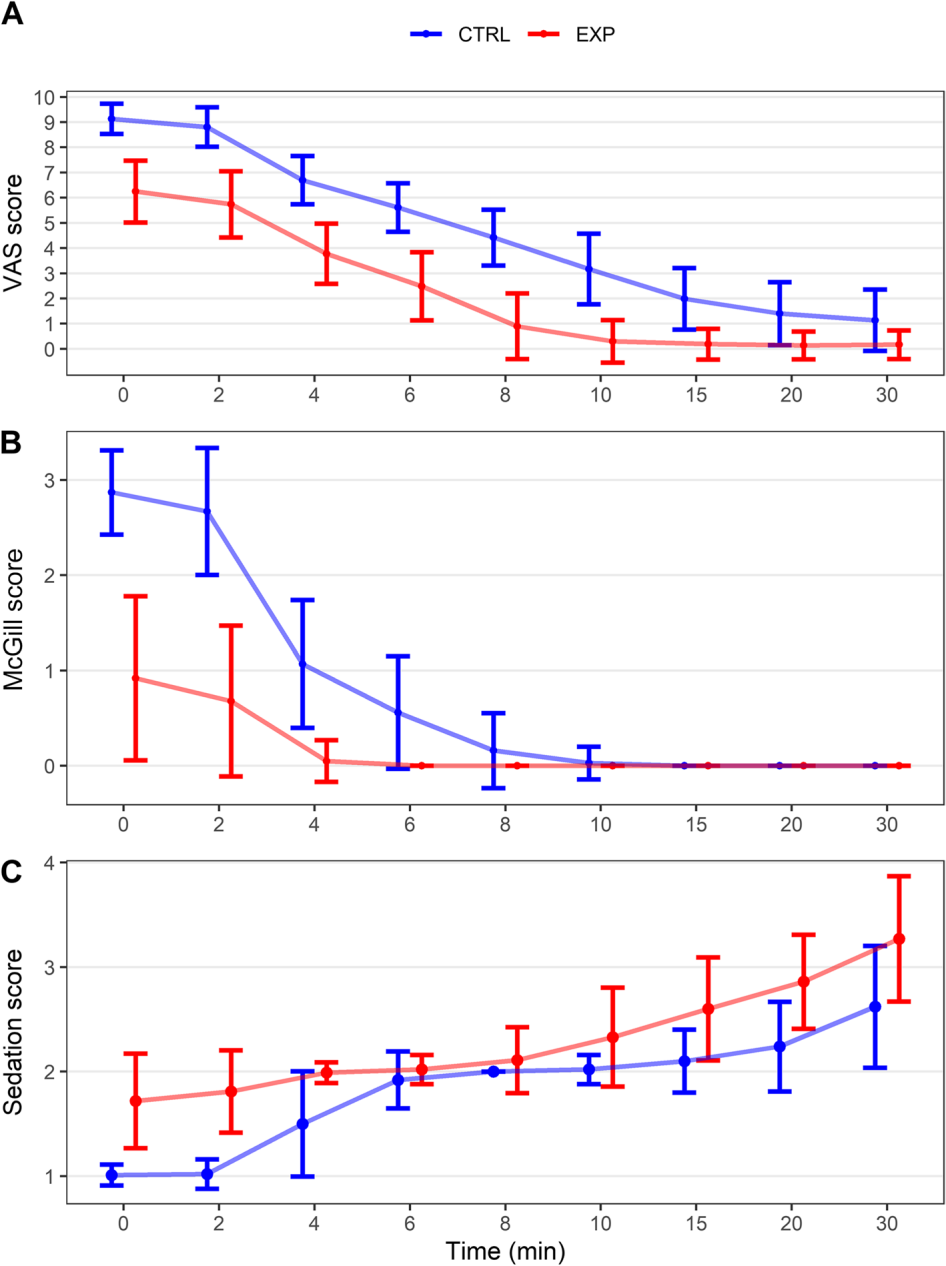
VAS scores, McGill scores, and sedation scores of the participants at different time points. **A** VAS scores. **B** McGill scores. **C** Sedation scores

With the exception of McGill scores at specific time points, indicators including pain intensity, maternal cooperation, VAS pain, and McGill scores at 0, 2, 4, 6, and 8 min were significantly lower in the experimental group than the CTRL group (Fig. 2A, B). The proportion of VAS scores ≤ 3 in the experimental group was notably higher at 4, 6, 8, and 10 min. Emotional score proportions were also significantly different, with a higher proportion of painlessness (score of 0) in the experimental group (except at 10, 15, 20, and 30 min) (Table 2).

**Table 2:** Primary outcomes of maternal delivery

Characteristic	CTRL		EXP		Statistics	*p*-value
	***N***	***N* (%) or mean ± SD**	***N***	***N* (%) or mean ± SD**		
Pain intensity score during epidural puncture	100	9.12 ± 0.61	100	6.25 ± 1.23	9519	< 0.001^a^
Maternal cooperation during epidural puncture	100	6.92 ± 1.68	100	3.12 ± 1.33	9524	< 0.001^a^
VAS pain at epidural						
0 min	100	9.13 ± 0.60	100	6.25 ± 1.23	9524	< 0.001^a^
2 min	100	8.81 ± 0.79	100	5.74 ± 1.32	9564	< 0.001^a^
4 min	100	6.70 ± 0.96	100	3.78 ± 1.19	9641.5	< 0.001^a^
6 min	100	5.61 ± 0.96	100	2.49 ± 1.35	9677.5	< 0.001^a^
8 min	100	4.42 ± 1.11	100	0.90 ± 1.31	9570	< 0.001a
10 min	100	3.17 ± 1.40	100	0.30 ± 0.85	9225.5	< 0.001^a^
15 min	100	1.99 ± 1.22	100	0.19 ± 0.61	8636	< 0.001^a^
20 min	100	1.40 ± 1.25	100	0.14 ± 0.55	7797	< 0.001^a^
30 min	100	1.14 ± 1.21	100	0.17 ± 0.57	7234	< 0.001^a^
VAS 0 min_group						
> 3	100	100 (100.00%)	100	100 (100.00%)		-
VAS 2 min_group						
> 3	100	100 (100.00%)	97	97 (97.00%)		0.246^c^
< = 3	0	0 (0.00%)	3	3 (3.00%)		
VAS 4 min_group						
> 3	100	100 (100.00%)	56	56 (56.00%)		< 0.001^c^
< = 3	0	0 (0.00%)	44	44 (44.00%)		
VAS 6 min_group						
> 3	97	97 (97.00%)	19	19 (19.00%)		< 0.001^c^
< = 3	3	3 (3.00%)	81	81 (81.00%)		
VAS 8 min_group						
> 3	80	80 (80.00%)	5	5 (5.00%)		< 0.001^c^
< = 3	20	20 (20.00%)	95	95 (95.00%)		
VAS 10 min_group						
> 3	42	42 (42.00%)	3	3 (3.00%)		< 0.001^c^
< = 3	58	58 (58.00%)	97	97 (97.00%)		
VAS 15 min_group						
> 3	6	6 (6.00%)	1	1 (1.00%)		0.118^c^
< = 3	94	94 (94.00%)	99	99 (99.00%)		
VAS 20 min_group						
> 3	2	2 (2.00%)	1	1 (1.00%)		1^c^
< = 3	98	98 (98.00%)	99	99 (99.00%)		
VAS 30 min_group						
> 3	2	2 (2.00%)	1	1 (1.00%)		1^c^
< = 3	98	98 (98.00%)	99	99 (99.00%)		
McGill score						
0 min	100	2.87 ± 0.44	100	0.92 ± 0.86	9455.5	< 0.001^a^
2 min	100	2.67 ± 0.67	100	0.68 ± 0.79	9428	< 0.001^a^
4 min	100	1.07 ± 0.67	100	0.05 ± 0.22	8910	< 0.001^a^
6 min	100	0.56 ± 0.59	100	0.00 ± 0.00	7550	< 0.001^a^
8 min	100	0.16 ± 0.39	100	0.00 ± 0.00	5750	< 0.001^a^
10 min	100	0.03 ± 0.17	100	0.00 ± 0.00	5150	0.083^a^
15 min	100	0.00 ± 0.00	100	0.00 ± 0.00		
20 min	100	0.00 ± 0.00	100	0.00 ± 0.00		
30 min	100	0.00 ± 0.00	100	0.00 ± 0.00		
McGill score 0 min_group					43.695	< 0.001^b^
0	1	1 (1.00%)	34	34 (34.00%)		
A	15	15 (15.00%)	6	6 (6.00%)		
B	2	2 (2.00%)	2	2 (2.00%)		
C	56	56 (56.00%)	49	49 (49.00%)		
D	26	26 (26.00%)	9	9 (9.00%)		
McGill score 2 min_group					63.921	< 0.001^b^
0	2	2 (2.00%)	49	49 (49.00%)		
A	15	15 (15.00%)	5	5 (5.00%)		
B	2	2 (2.00%)	2	2 (2.00%)		
C	55	55 (55.00%)	38	38 (38.00%)		
D	26	26 (26.00%)	6	6 (6.00%)		
McGill score 4 min_group					123.277	< 0.001^b^
0	18	18 (18.00%)	95	95 (95.00%)		
A	12	12 (12.00%)	1	1 (1.00%)		
B	1	1 (1.00%)	1	1 (1.00%)		
C	48	48 (48.00%)	0	0 (0.00%)		
D	21	21 (21.00%)	3	3 (3.00%)		
McGill score 6 min_group					68.456	< 0.001^c^
0	49	49 (49.00%)	100	100 (100.00%)		
A	8	8 (8.00%)	0	0 (0.00%)		
C	28	28 (28.00%)	0	0 (0.00%)		
D	15	15 (15.00%)	0	0 (0.00%)		
McGill score 8 min_group						
0	85	85 (85.00%)	100	100 (100.00%)	16.216	< 0.001^c^
A	1	1 (1.00%)	0	0 (0.00%)		
C	11	11 (11.00%)	0	0 (0.00%)		
D	3	3 (3.00%)	0	0 (0.00%)		
McGill score 10 min_group						
0	97	97 (97.00%)	100	100 (100.00%)	3.046	0.246^c^
C	2	2 (2.00%)	0	0 (0.00%)		
D	1	1 (1.00%)	0	0 (0.00%)		
McGill score 15 min_group						
0	100	100 (100.00%)	100	100 (100.00%)		
McGill score 20 min_group						
0	100	100 (100.00%)	100	100 (100.00%)		
McGill score 30 min_group						
0	100	100 (100.00%)	100	100 (100.00%)		

^a^*P*-values were calculated by applying Wilcoxon rank-sum test

^b^*P*-values were calculated by applying Pearson’s chi-squared test

^c^*P*-values were calculated by applying Fisher’s exact test

### Secondary outcome measures

**Fig. 3:**
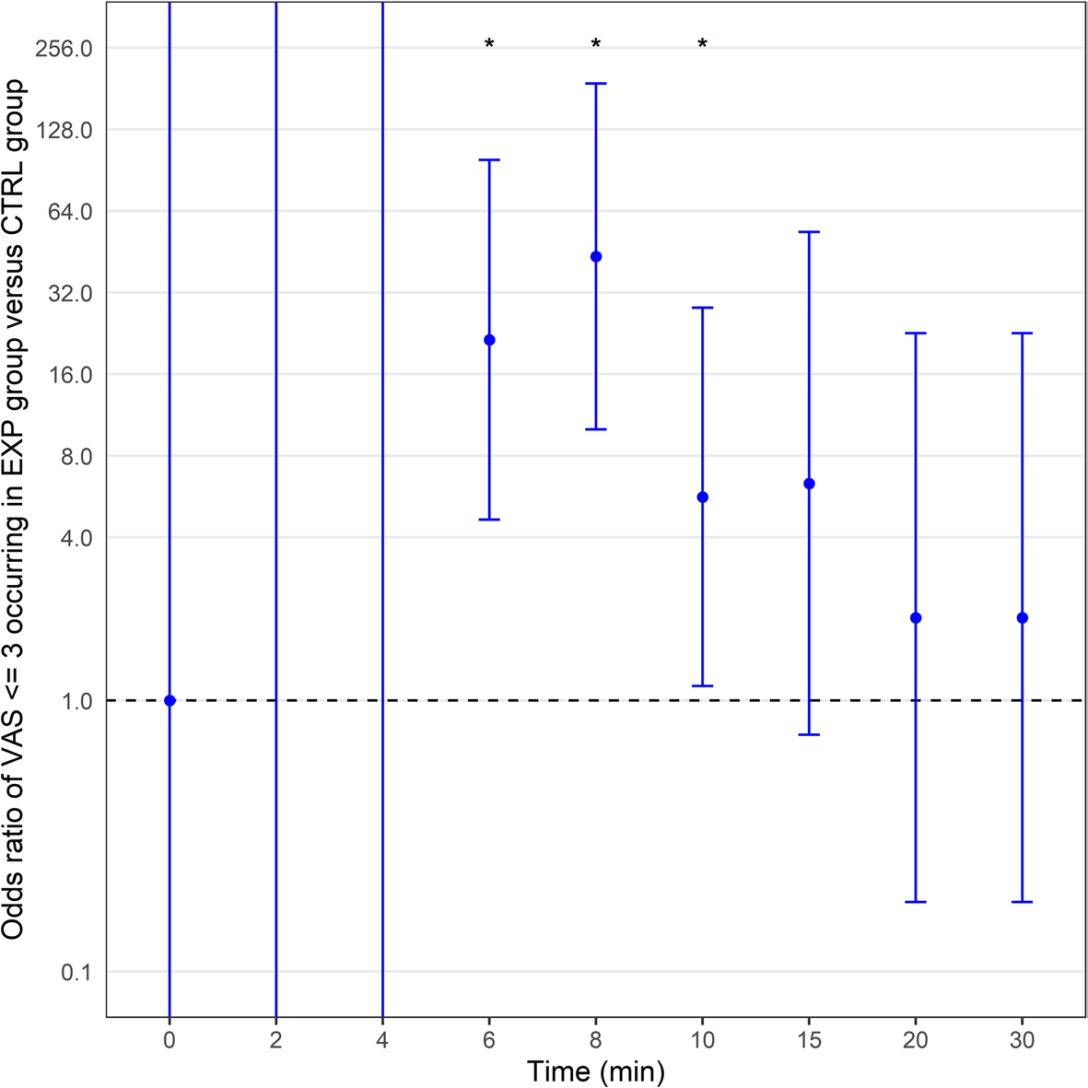
Odds ratio of *VAS* ≤ 3 occurring in the EXP group and CTRL group

Sedation scores at each time point were significantly higher in the experimental group than the CTRL group (Table 3 and Fig. 2C). The onset time of epidural analgesia was notably lower in the experimental group. No significant differences were observed in blood loss 24-h post-delivery and labor duration (Table 3). Neonatal indicators, except for umbilical arterial blood pH value, did not significantly differ between groups (Table 4). Moreover, we did not observe any instances of serious adverse events, such as respiratory depression, due to excessive sedation in parturients. Probability events analysis for pain intensity scores ≤ 6 during epidural puncture and maternal cooperation score ≤ 3 during epidural puncture highlighted grouping as an independent factor. The experimental group exhibited significantly higher probabilities for positive events in both cases (Tables 5 and 6). Further analysis of the probability of *VAS* ≤ 3 revealed significant differences at 6, 8, and 10 min, indicating greater efficacy of epidural analgesia in the experimental group during this time frame (Table 7 and Fig. 3). McGill scores of 0 at 4 min were significantly higher in the experimental group, suggesting an increased probability of painlessness (Table 8).

**Table 3:** Secondary outcomes of maternal delivery

Outcome	CTRL		EXP		Statistics	*p*-value*
	**N**	**Mean ± SD**	**N**	**Mean ± SD**		
Blood loss 24 h after delivery	100	332.30 ± 101.31	100	311.60 ± 73.04	5386	0.345
The first labor duration (min)	77	490.19 ± 276.99	76	491.87 ± 291.68	2951	0.929
The second labor duration (min)	77	37.58 ± 30.04	76	42.34 ± 38.77	2839	0.752
The third labor duration (min)	77	8.56 ± 4.34	76	9.36 ± 6.39	3016.5	0.73
Sedation score						
0 min	100	1.01 ± 0.10	100	1.72 ± 0.45	1450	< 0.001
2 min	100	1.02 ± 0.14	100	1.81 ± 0.39	1050	< 0.001
4 min	100	1.50 ± 0.50	100	1.99 ± 0.10	2550	< 0.001
6 min	100	1.92 ± 0.27	100	2.02 ± 0.14	4508	0.001
8 min	100	2.00 ± 0.00	100	2.11 ± 0.31	4450	< 0.001
10 min	100	2.02 ± 0.14	100	2.33 ± 0.47	3450	< 0.001
15 min	100	2.10 ± 0.30	100	2.60 ± 0.49	2500	< 0.001
20 min	100	2.24 ± 0.43	100	2.86 ± 0.45	2052	< 0.001
30 min	100	2.62 ± 0.58	100	3.27 ± 0.60	2482.5	< 0.001
Onset time of epidural analgesia	98	11.72 ± 3.29	99	5.58 ± 2.10	9282	< 0.001

^*^P-values were calculated by applying Wilcoxon rank-sum test

**Table 4:** Neonatal outcomes

Characteristic	CTRL		EXP		Statistics	*p*-value*
	***N***	**Mean ± SD**	***N***	**Mean ± SD**		
Umbilical arterial blood PH	91	7.29 ± 0.08	89	7.32 ± 0.07	3148	0.01
Umbilical arterial blood PCO_2_ (mmHg)	91	46.29 ± 10.66	89	42.34 ± 12.84	4625.5	0.1
BE.ecf (mmol/L)	91	− 5.28 ± 3.16	89	− 4.86 ± 3.25	3751	0.393
Weight (g)	99	3241.34 ± 394.27	100	3223.91 ± 428.64	4934.5	0.971
1-min Apgar	99	9.80 ± 0.61	100	9.91 ± 0.32	4682	0.216
5-min Apgar	99	9.97 ± 0.22	100	10.00 ± 0.00	4850	0.156

^*^P-values were calculated by applying Wilcoxon rank-sum test

**Table 5:** Odds ratio of pain intensity score during epidural puncture ≤ 6 occurring in EXP group versus CTRL group

Variable	Univariate		Multivariate	
	OR (95% *CI*)	*p*-value	OR (95% *CI*)	*p*-value
Group				
CTRL	1 (Reference)		1 (Reference)	
EXP	201.000 (26.839–1505.297)	< 0.001	201.000 (26.839–1505.297)	< 0.001
Age (years)	0.949 (0.877–1.026)	0.189		
Gestational weeks	0.926 (0.756–1.134)	0.457		
Parity				
1	1 (Reference)			
2	0.536 (0.246–1.169)	0.117		
3	1.929 (0.554–6.711)	0.302		
Height (m)	0.010 (0.000–5.008)	0.147		
Weight (kg)	0.967 (0.926–1.009)	0.125		
BMI (kg/m^2^)	0.964 (0.867–1.072)	0.5		
Mode of delivery				
Normal delivery	1 (Reference)			
Cesarean delivery	0.964 (0.459–2.022)	0.922		
Hypertensive disorders of pregnancy	2.114 (0.436–10.246)	0.353		
Gestational diabetes mellitus	1.495 (0.515–4.341)	0.46		
Cervical dilation (cm)	1.034 (0.668–1.601)	0.881		
VAS prior to inhalation of the nasal spray or saline	1.089 (0.517–2.294)	0.823		

Hosmer and Lemeshow test for multivariate logistic regression: chi-squared = 1.7688e-20, *df* = 8, *P*-value = 1

Odds ratios adjusted by using multivariate logistic regression with stepwise method

**Table 6:** Odds ratio of maternal cooperation during epidural puncture ≤ 3 occurring in EXP group versus CTRL group

Variable	Univariate		Multivariate	
	OR (95% *CI*)	*p*-value	OR (95% *CI*)	*p*-value
Group				
CTRL	1 (Reference)		1 (Reference)	
EXP	97.000 (28.214–333.487)	< 0.001	159.918 (34.239–746.928)	< 0.001
Age (years)	0.947 (0.878–1.023)	0.165		
Gestational weeks	1.018 (0.830–1.247)	0.866		
Parity				
1	1 (Reference)		1 (Reference)	
2	0.366 (0.169–0.797)	0.011	0.210 (0.049–0.900)	0.036
3	2.139 (0.560–8.174)	0.266	4.473 (0.411–48.626)	0.219
Height (m)	0.001 (0.000–0.438)	0.027	0.000 (0.000–9.344)	0.128
Weight (kg)	0.996 (0.956–1.037)	0.835		
BMI (kg/m^2^)	1.065 (0.961–1.180)	0.231		
Mode of delivery				
Normal delivery	1 (Reference)			
Cesarean delivery	0.738 (0.354–1.542)	0.42		
Hypertensive disorders of pregnancy	2.596 (0.536–12.566)	0.236		
Gestational diabetes mellitus	1.106 (0.416–2.944)	0.839		
Cervical dilation (cm)	0.777 (0.495–1.220)	0.274		
VAS prior to inhalation of the nasal spray or saline	1.188 (0.576–2.454)	0.641		

Hosmer and Lemeshow test for multivariate logistic regression: chi-squared = 4.6131, *df* = 8, *P*-value = 0.798

Odds ratios adjusted by using multivariate logistic regression with stepwise method

**Table 7:** Odds ratio of *VAS* ≤ 3 occurring in EXP group versus CTRL group

VAS ≤ 3	Comparison	Univariate		Multivariate	
		**OR (95% *CI*)**	***p*-value**	**OR (95% *CI*)**	***p*-value**
0 min	EXP vs CTRL	1.000 (0.000–Inf)	1	1.000 (0.000–Inf)	1
2 min	EXP vs CTRL	71,841,282.433 (0.000–Inf)	0.995	410,749.737 (0.000–Inf)	0.997
4 min	EXP vs CTRL	24,7001,869.090 (0.000–Inf)	0.986	13,297,787.216 (0.000–Inf)	0.992
6 min	EXP vs CTRL	137.842 (39.379–482.501)	< 0.001	21.414 (4.644–98.738)	< 0.001
8 min	EXP vs CTRL	76.000 (27.294–211.624)	< 0.001	43.439 (9.995–188.778)	< 0.001
10 min	EXP vs CTRL	23.414 (6.943–78.962)	< 0.001	5.637 (1.131–28.096)	0.035
15 min	EXP vs CTRL	6.319 (0.747–53.480)	0.091	6.319 (0.747–53.480)	0.091
20 min	EXP vs CTRL	2.020 (0.180–22.645)	0.568	2.020 (0.180–22.645)	0.568
30 min	EXP vs CTRL	2.020 (0.180–22.645)	0.568	2.020 (0.180–22.645)	0.568

Odds ratios adjusted by using multivariate logistic regression with stepwise method

**Table 8:** Odds ratio of emotional score = 0 occurring in EXP group versus CTRL group

McGill score = 0	Comparison	Univariate		Multivariate	
		**OR (95% *CI*)**	***p*-value**	**OR (95% *CI*)**	***p*-value**
0 min	EXP vs CTRL	51.000 (6.814–381.699)	< 0.001	0.262 (0.014–5.024)	0.374
2 min	EXP vs CTRL	47.078 (11.002–201.456)	< 0.001	0.697 (0.070–6.942)	0.758
4 min	EXP vs CTRL	86.556 (30.781–243.390)	< 0.001	12.100 (3.305–44.296)	< 0.001
6 min	EXP vs CTRL	-	-	-	-
8 min	EXP vs CTRL	-	-	-	-
10 min	EXP vs CTRL	-	-	-	-
15 min	EXP vs CTRL	-	-	-	-
20 min	EXP vs CTRL	-	-	-	-
30 min	EXP vs CTRL	-	-	-	-

Odds ratios adjusted by using multivariate logistic regression with stepwise method

### Correlation analysis

**Fig. 4:**
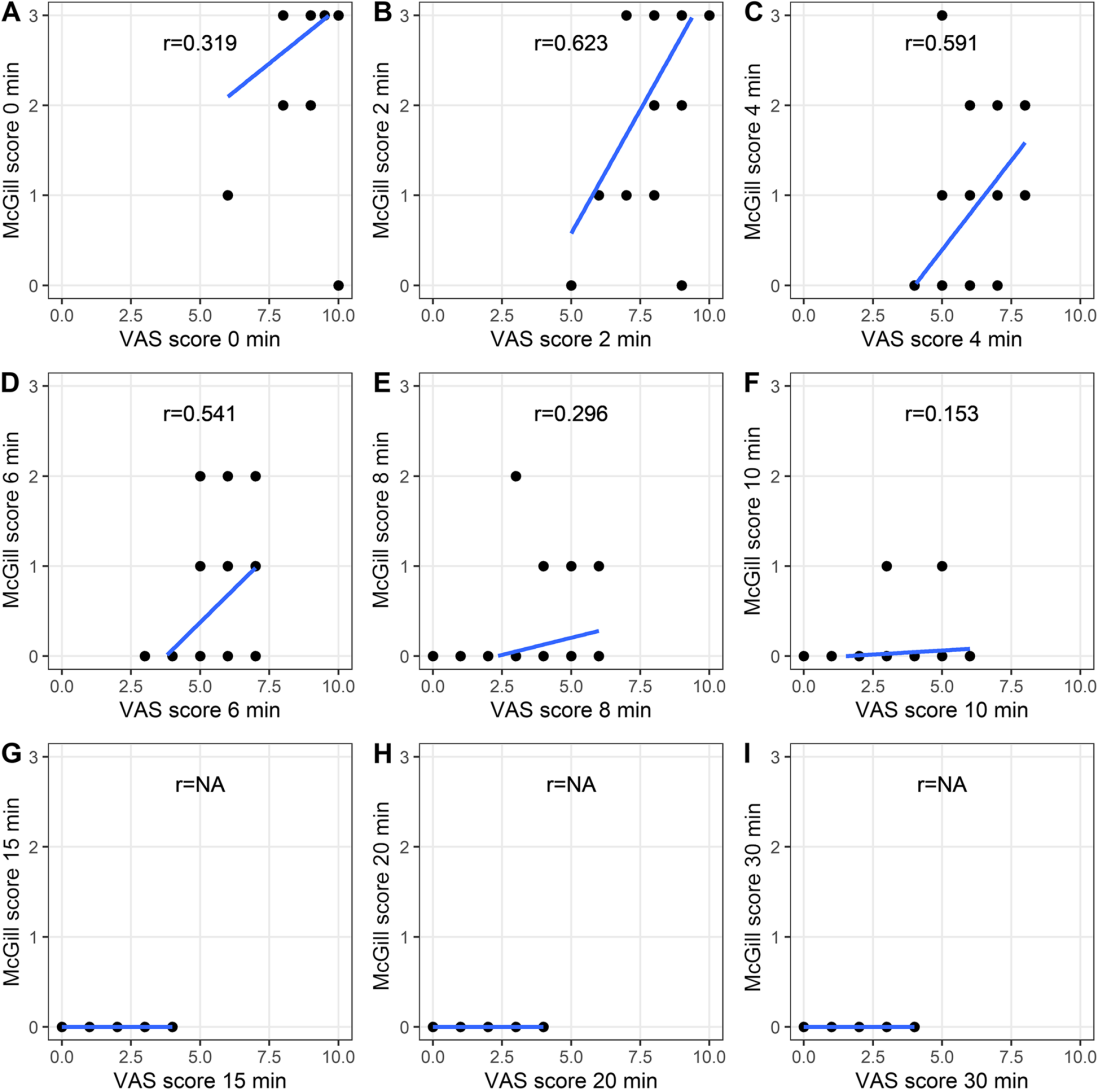
Correlation between the VAS score and emotional score in the CTRL group. **A**, **B**, **C**, **D**, **E**, **F**, **G**, **H**, **I** Scores at the 0-, 2-, 4-, 6-, 8-, 10-, 15-, 20-, and 30-min time points are shown, respectively

**Fig. 5:**
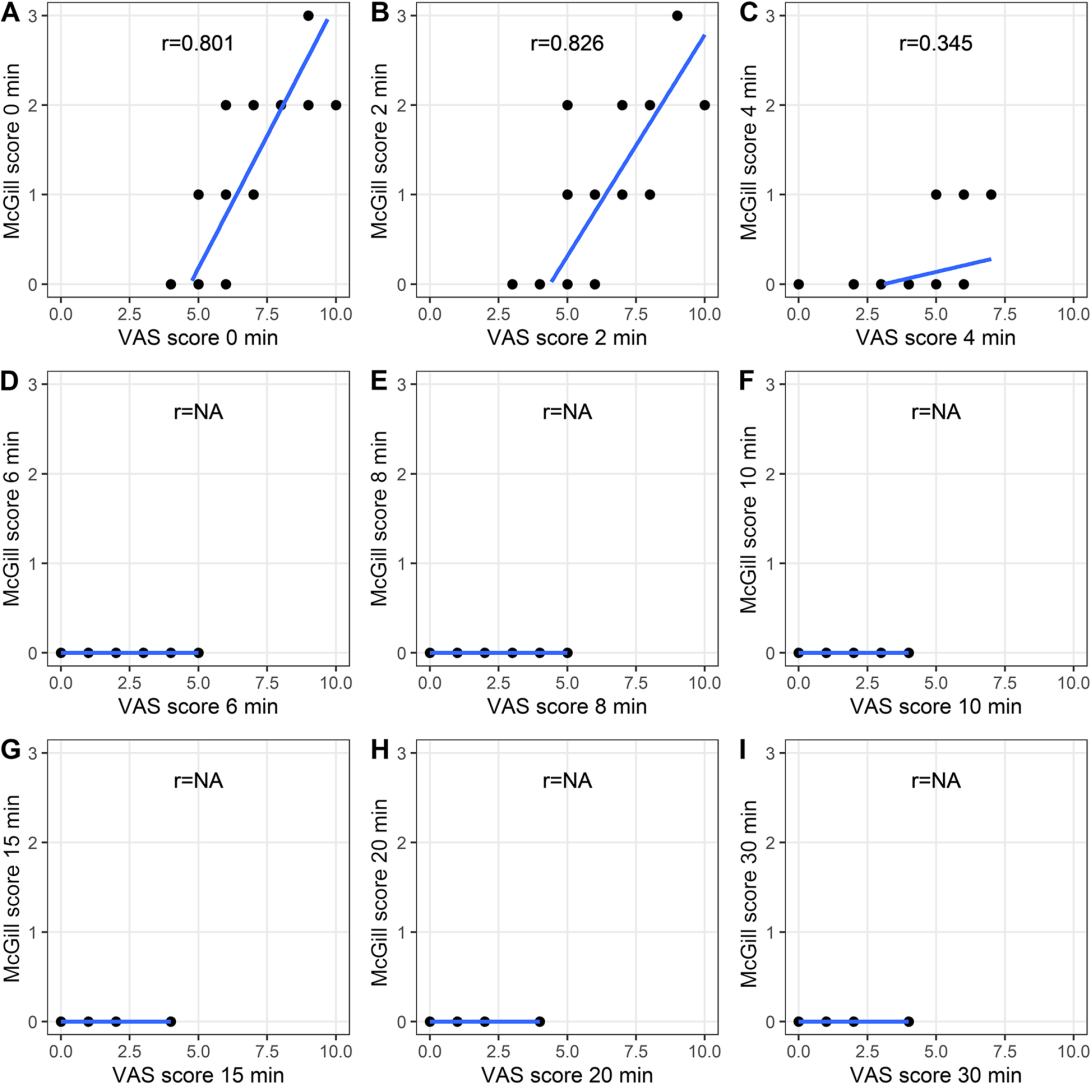
Correlation between the VAS score and emotional score in the EXP group. **A**, **B**, **C**, **D**, **E**, **F**, **G**, **H**, **I** Scores at the 0-, 2-, 4-, 6-, 8-, 10-, 15-, 20-, and 30-min time points are shown, respectively

**Fig. 6:**
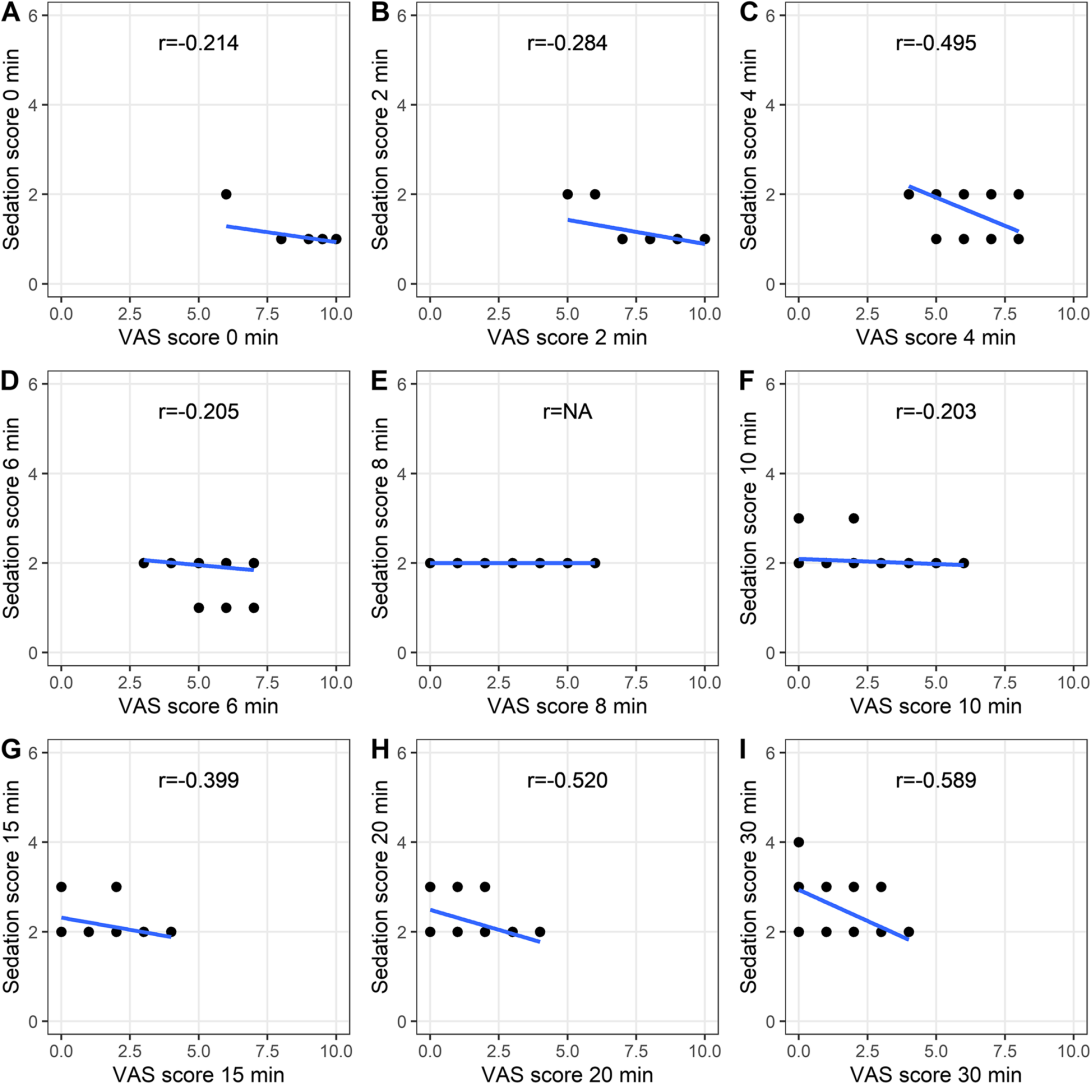
Correlation between the VAS score and sedation score in the CTRL group. **A**, **B**, **C**, **D**, **E**, **F**, **G**, **H**, **I** Scores at the 0-, 2-, 4-, 6-, 8-, 10-, 15-, 20-, and 30-min time points are shown, respectively

**Fig. 7:**
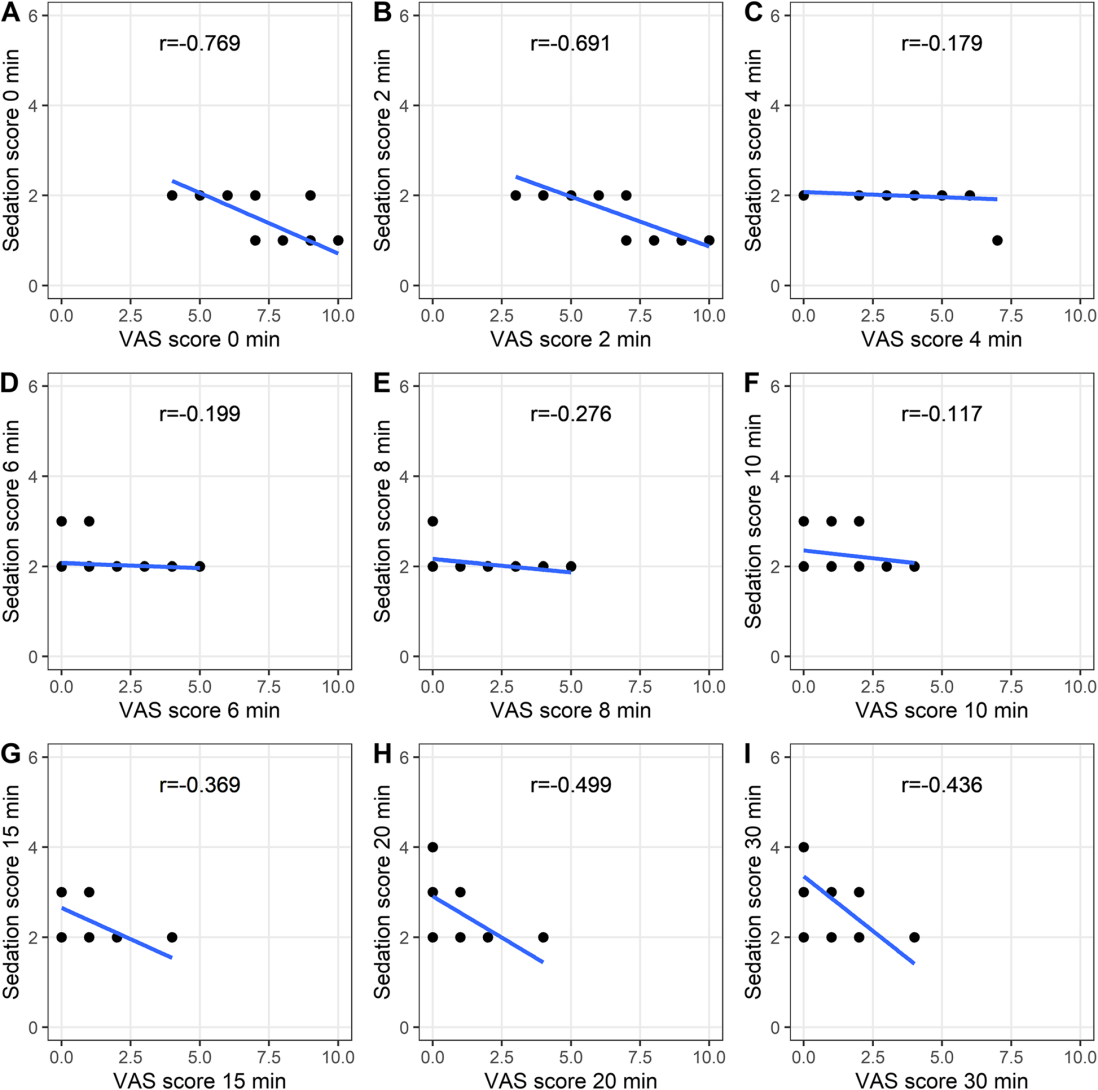
Correlation between the VAS score and sedation score in the EXP group. **A**, **B**, **C**, **D**, **E**, **F**, **G**, **H**, **I** Scores at the 0-, 2-, 4-, 6-, 8-, 10-, 15-, 20-, and 30-min time points are shown, respectively

Correlation analyses between VAS scores, McGill scores, and sedation scores at different time points revealed varying degrees of correlation in both groups. Notably, emotional and VAS scores were closely correlated in the CTRL group at 2, 4, and 6 min but weakly correlated at 0, 8, and 10 min. In the experimental group, the VAS score closely correlated with McGill scores at 0 and 2 min, with a weaker correlation at 4 min (Fig. 4 and Fig. 5). Additionally, correlation analyses between VAS scores and sedation scores indicated close relationships at specific time points in both groups (Fig. 6 and Fig. 7).

## Discussion

### Challenges in labor analgesia

Many parturients face difficulties cooperating during spinal labor analgesia due to missing the optimal timing or altering their preferences, posing risks during anesthesia puncture, and increasing the anesthesiologists’ workload (Angle et al. 2016). This study addresses this challenge by utilizing prenasal inhalation of butorphanol nasal spray combined with epidural anesthesia. This approach effectively alleviated maternal pain during epidural puncture, enhanced maternal cooperation, resulting in reduced pain scores during puncture, extended onset time for epidural labor analgesia, and improved corresponding affective scores.

### Safety and efficacy of butorphanol

Despite potential fetal sinusoidal effects, butorphanol’s safety during childbirth is affirmed, with no associated adverse outcomes (Welt 1985; Hatjis and Meis 1986). Studies highlight its placental transfer with epidural administration, showing no fetal respiratory depression (Yadav et al. 2018; Liu et al. 2021). Compared to potent opioids, butorphanol exhibits maternal safety, with lower adverse reaction rates than morphine (Pittman et al. 1980). Observations on neonates reveal no significant impact after intravenous administration (Shrestha et al. 2007; Wang et al. 2022). The butorphanol nasal spray in this study demonstrated satisfactory analgesic effects, rapid nasal onset, extended maintenance time, and low adverse reactions. Furthermore, no adverse reactions were noted, even in combination with epidural anesthesia. The butorphanol nasal spray utilized in this study offers non-invasiveness and ease of administration, and a single dose adequately meets the analgesic requirements. Patients exhibited good compliance, and our investigation affirmed rapid local absorption of butorphanol nasal spray, enhancing the parturient experience (Wermeling et al. 2005; Chu et al. 2004). Moreover, our findings indicated that butorphanol usage did not elevate adverse reaction rates associated with opioid drugs, with no instances of respiratory depression observed in parturients or fetuses. Hence, we assert that compared to other potent opioid medications, butorphanol administration in parturients boasts enhanced safety, aligning with prior research outcomes.

### Clinical outcomes and analgesic synergy

In the experimental group, pre-analgesia with butorphanol nasal spray before epidural puncture resulted in significantly lower VAS scores and McGill scores during puncture, indicating enhanced maternal comfort and cooperation. Throughout the study, the experimental group consistently showed lower VAS scores and McGill scores than the CTRL group, affirming the sustained efficacy of butorphanol. The onset time for epidural analgesia was notably shorter in the experimental group, possibly indicating a synergistic effect between butorphanol and intraspinal epidural analgesia, resulting in quicker onset times for analgesia scores ≤ 3.

### Analgesic and emotional impact

Previous research suggests that combining butorphanol with opioid receptor agonists enhances analgesic effects while mitigating side effects like respiratory depression (Yin and Zhang 2019; Chaurasia et al. 2017). This study supports these findings, indicating a dual benefit in reducing both subjective pain and associated adverse emotional experiences. Maternal discomfort during epidural puncture improved from severe to moderate or mild, contributing to a more positive overall experience for the parturients. Considering the prevailing perspective favoring early anesthesia administration to mitigate maternal pain onset during labor initiation, our study focused on limiting butorphanol use by parturients at the onset of labor to enhance their cooperation during epidural anesthesia. However, it is worth noting that for parturients experiencing rapid labor progression or in advanced labor stages requiring analgesia, the intranasal butorphanol method might not be suitable, posing a limitation to our study. Future research endeavors could involve designing additional clinical trials to investigate analgesic strategies throughout the entire labor process.

### Neonatal safety and blood gas analysis

Analysis of neonatal umbilical cord arterial blood gas revealed no significant differences in most indicators between the two groups. The pH value in the experimental group was superior, suggesting potential benefits related to excellent analgesia, lower McGill scores, and good sedation. The early use of butorphanol nasal spray demonstrated effectiveness, and no notable adverse effects were observed in neonates during the long-term observation.

### Study limitations and future considerations

While the study’s pain and emotional rating scales (VAS and emotional score) have been widely used in previous research (Tang et al. 2021; Peker and Polat 2021), accounting for potential influencing factors such as patient mental state and cultural background remains a complex challenge. Future research could explore further factors affecting patient experiences during anesthesia procedures, aiming to enhance the reliability and validity of findings. Additionally, our primary outcome regarding maternal cooperation during the placement of epidural anesthesia was assessed by anesthesiologists using subjective judgment combined with the VAS. The lack of relatively objective indicators to demonstrate maternal cooperation represents a limitation of our study. In the future, we hope to develop a scale that can objectively and specifically reflect maternal cooperation.

## Conclusion

In conclusion, the combined use of butorphanol nasal spray with epidural analgesia effectively reduces maternal pain during labor, enhances cooperation, and demonstrates synergistic effects with opioid agonists, improving analgesic efficacy while minimizing side effects. The study affirms the safety and efficacy of this combined approach, indicating its potential as a beneficial option for maternal analgesia during labor.

## Data Availability

No datasets were generated or analysed during the current study.

## Abbreviations

CTRL group: Control group
EXP group: Experimental group
BE-ecf: Base excess of extracellular fluid
VAS: Visual analogue scale
ASA: American Society of Anesthesiologists
PCO_2_: Partial pressure CO_2_
RCT: Randomized controlled trial
PCEA: Patient-controlled epidural analgesia
PCA: Patient-controlled analgesia
NBP: Noninvasive blood pressure
HR: Heart rate
SpO2: Oxygen saturation
SF-MPQ: Short Form of the McGill Pain Questionnaire
